# Relative abundance of nitrogen cycling microbes in coral holobionts reflects environmental nitrate availability

**DOI:** 10.1098/rsos.201835

**Published:** 2021-06-02

**Authors:** Arjen Tilstra, Florian Roth, Yusuf C. El-Khaled, Claudia Pogoreutz, Nils Rädecker, Christian R. Voolstra, Christian Wild

**Affiliations:** ^1^ Marine Ecology Department, Faculty of Biology and Chemistry, University of Bremen, Bremen, Germany; ^2^ Red Sea Research Center, King Abdullah University of Science and Technology, Thuwal, Kingdom of Saudi Arabia; ^3^ Baltic Sea Centre, Stockholm University, Stockholm, Sweden; ^4^ Tvärminne Zoological Station, Faculty of Biological and Environmental Sciences, University of Helsinki, Helsinki, Finland; ^5^ Department of Biology, University of Konstanz, Konstanz, Germany; ^6^ Laboratory for Biological Geochemistry, School of Architecture, Civil and Environmental Engineering, École Polytechnique Fédérale de Lausanne (EPFL), Lausanne, Switzerland

**Keywords:** coral reefs, Scleractinia, seasonality, denitrification, dinitrogen fixation, diazotrophy

## Abstract

Recent research suggests that nitrogen (N) cycling microbes are important for coral holobiont functioning. In particular, coral holobionts may acquire bioavailable N via prokaryotic dinitrogen (N_2_) fixation or remove excess N via denitrification activity. However, our understanding of environmental drivers on these processes *in hospite* remains limited. Employing the strong seasonality of the central Red Sea, this study assessed the effects of environmental parameters on the proportional abundances of N cycling microbes associated with the hard corals *Acropora hemprichii* and *Stylophora pistillata.* Specifically, we quantified changes in the relative ratio between *nirS* and *nifH* gene copy numbers, as a proxy for seasonal shifts in denitrification and N_2_ fixation potential in corals, respectively. In addition, we assessed coral tissue-associated Symbiodiniaceae cell densities and monitored environmental parameters to provide a holobiont and environmental context, respectively. While ratios of *nirS* to *nifH* gene copy numbers varied between seasons, they revealed similar seasonal patterns in both coral species, with ratios closely following patterns in environmental nitrate availability. Symbiodiniaceae cell densities aligned with environmental nitrate availability, suggesting that the seasonal shifts in *nirS* to *nifH* gene abundance ratios were probably driven by nitrate availability in the coral holobiont. Thereby, our results suggest that N cycling in coral holobionts probably adjusts to environmental conditions by increasing and/or decreasing denitrification and N_2_ fixation potential according to environmental nitrate availability. Microbial N cycling may, thus, extenuate the effects of changes in environmental nitrate availability on coral holobionts to support the maintenance of the coral–Symbiodiniaceae symbiosis.

## 1. Introduction

The oligotrophic nature of coral reefs requires an efficient use and recycling of the available nutrients within the ecosystem, including by their main engineers, scleractinian corals. As such, corals consist not only of the animal host alone but additionally harbour a diverse range of eukaryotic and prokaryotic microorganisms [1], rendering it a so-called ‘holobiont’. Many of these coral-associated microorganisms aid in nutrient (re)cycling [2,3]. Nitrogen (N) is an essential macronutrient, the availability of which often being the controlling factor for primary production (i.e. the fixation of dissolved inorganic carbon (DIC) through photosynthesis performed by the Symbiodiniaceae) in coral holobionts [4,5]. Despite the importance of N for coral holobionts, *in hospite* limitation of N is crucial for maintaining the symbiosis between the coral animal and the photosynthetic algal symbionts of the family Symbiodiniaceae [6]. Translocation of photosynthates by Symbiodiniaceae, i.e. the main supply of organic C for the coral host [7], is optimal under N limitation [8–10]. The interruption of N limitation may, thus, lead to the cessation of photosynthate translocation, which may ultimately lead to the breakdown of the coral–Symbiodiniaceae symbiosis due to increased bleaching susceptibility [9,11,12]. Thus, the cycling of N is critical for understanding coral holobiont functioning [9].

The environmental availability of N fluctuates in coral reef environments. This may include natural fluctuations, e.g. seasonality in N availability [13–16], as well as anthropogenic N inputs [17]. In this sense, coral-associated microbes, in particular prokaryotes, may play an integral role in coral holobiont N cycling. On the one hand, diazotrophs, prokaryotes capable of fixing atmospheric dinitrogen (N_2_), may provide the coral holobiont with de novo bioavailable N in the form of ammonium in times of environmental N scarcity [18–20]. On the other hand, microbes capable of denitrification, i.e. the chemical reduction of nitrate to N_2_, may play a putative role in alleviating the coral holobiont from excess N [21,22]. It was hypothesized that high denitrification rates may maintain N limitation for Symbiodiniaceae, and, as a result, may potentially support the functioning of the coral–Symbiodiniaceae symbiosis [9,11]. To this end, the presence of denitrifiers in coral holobionts was first reported in the late 2000s [23,24], but Tilstra *et al.* [21] only recently demonstrated that denitrification indeed constitutes an active metabolic pathway present in coral holobionts from the oligotrophic central Red Sea.

Taken together, microbial N cycling contributes to N availability for the coral holobiont. However, our understanding of how abiotic and biotic factors affect N cycling properties in corals remains poorly understood. Making use of the pronounced seasonality of the central Red Sea, the present study aimed to (i) assess patterns in the abundance of denitrifiers (approximated via *nirS* gene copy numbers) in relation to diazotrophs (approximated via *nifH* gene copy numbers) in a seasonal resolution (herein referred to as *nirS* to *nifH* gene abundance ratios); and (ii) identify environmental parameters potentially driving the observed seasonal patterns. Due to the potential stimulating or suppressing effects of dissolved inorganic N (DIN) on denitrification [22,25] and diazotrophy [22,26–29], respectively, we hypothesized that the seasonal patterns of *nirS* to *nifH* gene abundance ratios in coral holobionts would be mostly affected by DIN, i.e. nitrate, nitrite and/or ammonium concentrations.

## 2. Material and methods

### 2.1. Sample collection

**Figure 1.**
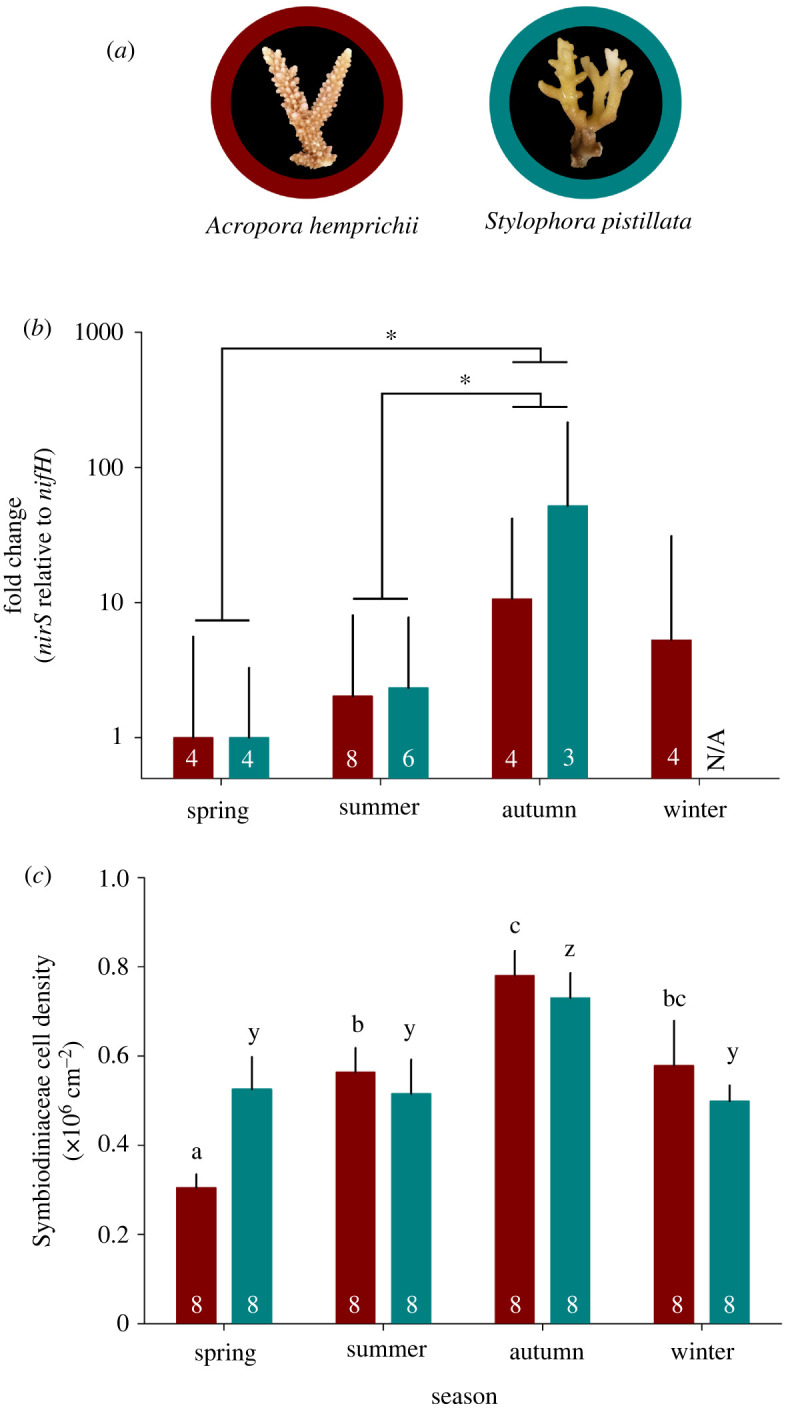
Patterns of *nirS* to *nifH* gene abundance ratios and Symbiodiniaceae cell densities associated with two central Red Sea hard coral species across four seasons. (*a*) Representative photographs of investigated species, (*b*) fold change of *nirS* to *nifH* gene abundance ratios and (*c*) Symbiodiniaceae cell densities. Fold changes were calculated in relation to spring, during which both species exhibited the lowest *nirS* to *nifH* gene abundance ratios; bars indicate the mean; error bars indicate upper confidence intervals (+1 s.e.). Numbers in the bars represent the sample size (*n*). Within plot (*b*), asterisks indicate significant differences between seasons (pair-wise PERMANOVA, *p < 0.05). Within plot (*c*), different letters above error bars indicate statistically significant differences per species between seasons (pair-wise PERMANOVA, *p* < 0.05). N/A = not available.

Two common species of hard coral (figure 1*a*), i.e. *Acropora hemprichii* (Acroporidae) and *Stylophora pistillata* (Pocilloporidae), were collected over four seasons: April 2017 (spring), August 2017 (summer), November 2017 (autumn) and January 2018 (winter). Corals were collected at approx. 5 m water depth at the semi-exposed side of the inshore reef Abu Shosha (22°18′15″ N, 39°02′56″ E) located in the Saudi Arabian central Red Sea. Sailing permits were issued by the Saudi Arabian Coastguard Authority to the sites that included coral collection. During each season, eight fragments of each coral species were collected from spatially separated colonies (distance apart greater than 10 m) to ensure genetic diversity. Immediately after collection, fragments were flash frozen in liquid N aboard the research vessel. Subsequently, fragments were transported to the laboratories of the King Abdullah University of Science and Technology and stored at −80°C until further processing.

### 2.2. DNA extraction and quantitative PCR

Coral tissues were separated from the skeleton by pressurized air. DNA was extracted from 100 µl of the resulting tissue slurry using the Qiagen DNeasy Plant Mini Kit (Qiagen, Germany) according to the manufacturer's instructions. Extracted DNA was stored at −20°C until qPCR assays were performed. Quantitative PCRs (qPCRs) were carried out according to Tilstra *et al.* [21]. Briefly, relative copy numbers of the functional genes *nirS* and *nifH* were used as a proxy for denitrification and diazotrophy, respectively, as implemented previously [20,21,30]. qPCR assays were performed in technical triplicates for each biological replicate (i.e. coral fragment), which were averaged before statistical analysis. Each assay contained 9 µl reaction mixture and 1 µl DNA template (input adjusted to approx. 3 ng DNA µl^−1^). The reaction mixture contained 5 µl Platinum SYBR Green qPCR Master Mix (Invitrogen, Carlsbad, CA, United States), 0.2 µl of each primer (10 µM), 0.2 µl of ROX dye and 3.4 µl of nuclease-free water (see table 1 for primers used). *NirS* to *nifH* gene abundance ratios were determined by normalizing against the *nifH* gene. The thermal cycling protocol was 50°C for 2 min, 95°C for 2 min, 50 cycles of 95°C for 30 s, 51°C for 1 min, 72°C for 1 min and a 72°C extension cycle for 2 min. Amplification specificity was determined by adding a dissociation step (melting curve analysis). All assays were performed on the ABI 7900HT Fast real-time PCR System (Applied Biosystems, CA, USA). Standard calibration curves were run simultaneously covering eight orders of magnitude (10^1^–10^8^ copies of template per assay each for the *nirS* and *nifH* gene). The qPCR efficiency (E) of the primer pairs was 86% and 87%, respectively, calculated according to the equation E = [10^(−1/slope)^−1]. *nirS* to *nifH* gene abundance ratios were calculated as 2^(−ΔΔCt)^ against *nifH* Ct values using the season with the lowest relative abundances as the reference [33].

**Table 1.** Selected primers used for amplification.

target gene	primer	nucleotide sequence (5′ → 3′)	reference
*nirS*	cd3aF	GTSAACGTSAAGGARACSGG	[31]
	R3cd	GASTTCGGRTGSGTCTTGA	
*nifH*	F2	TGYGAYCCIAAIGCIGA	[32]
	R6	TCIGGIGARATGATGGC	

### 2.3. Symbiodiniaceae cell densities

A further aliquot of the tissue slurry used for DNA extraction was used to obtain cell densities of Symbiodiniaceae. Tissue slurry aliquots were homogenized, diluted at a ratio of 3 : 1 or 5 : 1, and Symbiodiniaceae cells were subsequently counted using a Neubauer-improved haemocytometer on a light microscope with HD camera (Zeiss, Germany). Resulting photographs were analysed using the Cell Counter Notice in ImageJ software (National Institutes of Health, USA). Cell counts for tissue slurries for each individual coral were done in duplicates and subsequently averaged. Finally, to obtain cell densities of Symbiodiniaceae per unit area of coral tissue, cell counts were normalized to the coral surface area, which was calculated using cloud-based three-dimensional models of samples (Autodesk Remake v. 19.1.1.2) [34,35].

### 2.4. Environmental parameters

Environmental data, i.e. temperature, light intensity (photosynthetically active radiation [PAR = 400–700 nm], salinity, dissolved oxygen (DO), nitrate, nitrite, ammonium, dissolved inorganic phosphorus (DIP = [phosphate]) and dissolved organic carbon (DOC), were described and published previously in Roth *et al*. [14,36] and were reanalysed for the purposes of the present study.

The temperature was measured continuously with data loggers (Onset HOBO Water Temperature Pro v. 2 Data Logger U22–001; accuracy: ± 0.21°C) one month prior and within the month of sampling on a 30 min interval. Light availability (lux) was measured with data loggers (Onset HOBO Pendant UA-002-64; spectral detection range 150–1200 nm) for 3 full days every month and converted to photosynthetically active radiation (PAR = 400–700 nm) using a conversion factor of 51.8 as outlined by Roth *et al*. [14]. The conversion factor was obtained by inter-calibrating the lux readings (i.e. from the Onset HOBO Pendant) with data obtained from a parallel deployment of a PAR sensor (LI-COR LI-1500 quantum sensor) during 4 h of daylight. Both readings correlated (*r*^2^ = 0.91) and the obtained conversion factor was 51.8. Salinity was measured on three days every month using a conductivity measuring cell (TetraCon®, 925, WTW, accuracy: ± 0.5% of value, internal conversion to salinity). DO was quantified on 2 days within the month of sampling by taking the average of eight autonomous recording DO and temperature sensors (HOBO U26; temperature corrected and salinity adjusted) that were deployed at 5 m water depth within a radius of 50 m of the sampling site. Seawater samples were taken in triplicates from directly above the reef at the sampling site on 3 days monthly or bimonthly, i.e. one month prior to sampling and/or the month of sampling, to measure (in)organic nutrients. Nitrate, nitrite and DIP were measured photometrically, while ammonium was measured fluorometrically. DIN was calculated as [nitrate] + [nitrite] + [ammonium]. Subsamples for DOC were filtered through 0.2 μm Millipore® polycarbonate filters into pre-combusted (450°C, 4.5 h) acid-washed amber glass vials (Wheaton) with Teflon-lined lids, and samples were subsequently acidified with H_3_PO_4_ until reaching pH 1–2. Samples were kept in the dark at 4°C until further analysis by high-temperature catalytic oxidation using a total organic carbon analyser (Shimadzu, TOC-L). To monitor the accuracy of DOC concentration measurements, we used reference material of deep-sea carbon (42–45 µmol C l^−1^) and low-carbon water (1–2 µmol C l^−1^).

### 2.5. Statistical analyses

To assess seasonality, data were analysed using non-parametric permutational multivariate analysis of variance (PERMANOVA) using PRIMER-E version 6 software [37] with the PERMANOVA + add on [38]. To test for differences in *nirS* and *nifH* gene abundance ratios and Symbiodiniaceae cell densities between seasons, two-factorial univariate PERMANOVAs were performed with season and coral species as main factors based on Euclidian distances of square-root transformed data [39], while one-factorial univariate PERMANOVAs were performed with the season as the main factor for environmental parameters based on Euclidian distances of normalized data [39]. Type III (partial) sum of squares was therefore used with an unrestricted permutation of raw data (999 permutations), and PERMANOVA pair-wise tests with parallel Monte Carlo tests (with Bonferroni corrected *p*-values) were carried out when significant differences were found, to account for multiple comparisons.

Pearson product-moment correlation tests were performed to identify correlations between *nirS* to *nifH* gene abundance ratios, Symbiodiniaceae cell density and environmental variables. Salinity and DIP were omitted from the analyses as differences were assumed to have no ecological significance. In addition, nitrite and ammonium were omitted from the analyses as there were no significant differences between seasons (electronic supplementary material, table S1). Finally, linear regression analysis was used to assess a potential statistical relationship between *nirS* to *nifH* gene abundance ratios and Symbiodiniaceae cell density over all seasons. All values are given as mean ± s.e.

## 3. Results

### 3.1. *nirS* to *nifH* gene abundance ratios

The extracted DNA was of varying quality, and amplification was not possible in some samples, resulting in varying levels of replicates for each species and season. The resulting sample size is indicated within figure 1*b*.

Lowest *nirS* (as a proxy for denitrification) to *nifH* (as a proxy for N_2_ fixation) gene abundance ratios were observed during the spring season, hence gene abundance ratios for other seasons were calculated as fold changes in relation to spring (figure 1*b*). In summer, gene abundance ratios increased approximately twofold for both species, while they increased approximately 11-fold during autumn for *A. hemprichii* and approximately 52-fold for *S. pistillata* (figure 1*b*). During winter, gene abundance ratios were approximately fivefold higher in *A. hemprichii* compared with spring (no data available for *S. pistillata* for this season) (figure 1*b*). There was no interactive effect of season and species on gene abundance ratios (PERMANOVA, pseudo-*F* = 0.13, *p* = 0.898; electronic supplementary material, table S1). However, there was an effect of season (PERMANOVA, pseudo-*F* = 3.27, *p* = 0.039; electronic supplementary material, table S1) and species (PERMANOVA, pseudo-*F* = 6.22, *p* = 0.022; electronic supplementary material, table S1) on gene abundance ratios. Indeed, gene abundance ratios were higher during autumn compared with spring (pair-wise PERMANOVA, *t* = 3.22, *p* = 0.015) and summer (pair-wise PERMANOVA, *t* = 2.67, *p* = 0.015). See electronic supplementary material, table S2 for full details of pair-wise comparisons.

### 3.2. Symbiodiniaceae cell densities

Cell densities of Symbiodiniaceae varied more strongly between seasons in *A. hemprichii* compared with *S. pistillata* (electronic supplementary material, table S1; figure 1*c*). Cell densities for *A. hemprichii* were lowest in spring (0.31 ± 0.03 × 10^6^ cells cm^−2^) and significantly increased during summer (0.56 ± 0.05 × 10^6^ cells cm^−2^; pair-wise PERMANOVA, *t* = 4.29, *p* = 0.001) (figure 1*c*). Subsequently, cell densities significantly increased in autumn (0.78 ± 0.06 × 10^6^ cells cm^−2^; pair-wise PERMANOVA, *t* = 2.78, *p* = 0.021), but returned to densities similar to summer, during winter (0.59 ± 0.10 × 10^6^ cells cm^−2^) (figure 1*c*). Cell densities of Symbiodiniaceae in tissues of *S. pistillata* were similar during spring, summer and winter (0.53 ± 0.07, 0.52 ± 0.08, 0.50 ± 0.04 × 10^6^ cells cm^−2^, respectively) (figure 1*c*). However, densities during autumn were significantly higher compared with the other seasons (0.73 ± 0.06 × 10^6^ cells cm^−2^; pair-wise PERMANOVA, *p* < 0.05) (figure 1*c*). See electronic supplementary material, table S2 for full details of pair-wise comparisons.

### 3.3. Environmental parameters

**Figure 2.**
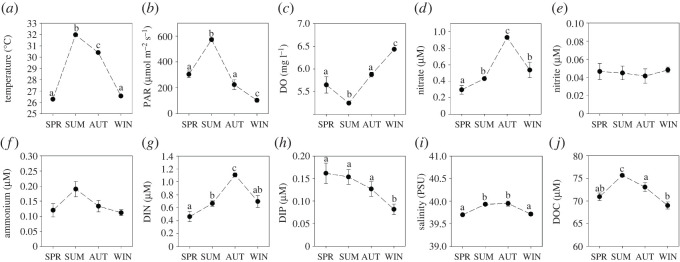
Means (±s.e.) of environmental parameters measured over four seasons. (*a*) Temperature, (*b*) PAR, (*c*) DO, (*d*) nitrate, (*e*) nitrite, (*f*) ammonium, (*g*) dissolved inorganic nitrogen (DIN = [nitrate] + [nitrite] + [ammonium]), (*h*) dissolved inorganic phosphorus (DIP = [phosphate]), (*i*) salinity and (*j*) DOC. Different letters above error bars indicate significant differences between seasons within each plot (*p* < 0.05). SPR = spring; SUM = summer; AUT = autumn; WIN = winter. Data were extracted from Roth *et al*. [14,36] and re-analysed for the purpose of this study.

Several environmental parameters exhibited marked seasonal fluctuations (figure 2; electronic supplementary material, table S1). Temperature and PAR increased from spring to the summer season when both parameters were at their highest (31.99 ± 0.01°C and 573 ± 13 µmol m^−2^ s^−1^, respectively) (figure 2*a* and *b*). DO was lowest in summer (5.25 ± 0.03 mg l^−1^) and highest in winter (6.44 ± 0.03 mg l^−1^) (figure 2*c*). Nitrate was highest during the autumn season (0.93 ± 0.02 µM) and lowest during the spring season (0.30 ± 0.05 µM) (figure 2*d*). Nitrite remained stable throughout all seasons (0.05 ± 0.01 µM) (figure 2*e*). Ammonium remained stable throughout all seasons (0.14 ± 0.02 µM) (figure 2*f*). DIN followed the same pattern as nitrate being highest during the autumn season (1.11 ± 0.03 µM) and lowest during the spring season (0.46 ± 0.08 µM) (figure 2*g*; electronic supplementary material, figure S1). DIP was stable from spring until autumn but decreased during winter (0.08 ± 0.01 µM) (figure 2*h*). Salinity remained relatively stable throughout the period of study (39.85 ± 0.02 PSU) (figure 2*i*). DOC was highest in summer (75.66 ± 0.40 µM) and lowest in spring (70.93 ± 0.82 µM) and winter (68.99 ± 0.77 µM) (figure 2*j*). See electronic supplementary material, table S2 for full details of pair-wise comparisons.

### 3.4. Correlation analyses

Due to the lack of a significant interaction between season and species in *nirS* to *nifH* gene abundance ratios (electronic supplementary material, table S1), data for both species were pooled for correlation analyses.

**Figure 3.**
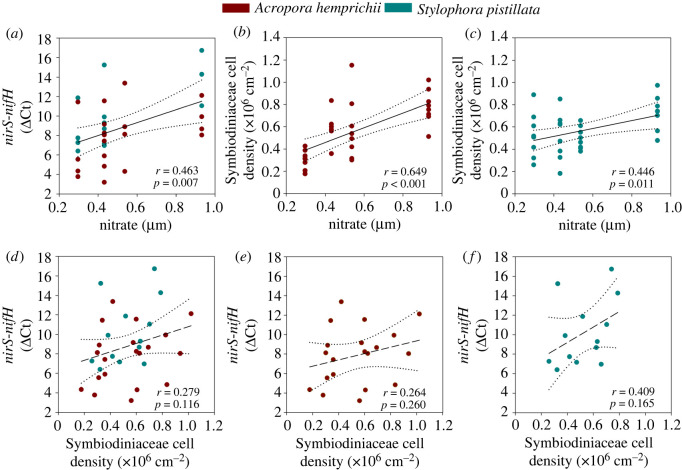
Pearson product-moment correlation analyses for (*a*) ΔCt of *nirS*-*nifH* against environmental nitrate concentrations pooled for both coral species, cell densities of Symbiodiniaceae against environmental nitrate concentrations for (*b*) *A. hemprichii* and (*c*) *S. pistillata* and linear regression analyses for (*d*) ΔCt of *nirS*-*nifH* against cell densities of Symbiodiniaceae pooled for both coral species, for (*e*) *A. hemprichii* and (*f*) *S. pistillata*. *r* = Pearson coefficient. Best-fit linear regression lines ± 95% confidence intervals (dotted lines) are solid when a significant relationship was established; lines are dashed when not significant.

The most significant correlation for both species' *nirS* to *nifH* gene abundance ratios was with nitrate (Pearson product-moment correlation, *r* = 0.463, *p* = 0.007; figure 3*a*; electronic supplementary material, table S3). Symbiodiniaceae cell densities also correlated most significantly with nitrate for both *A. hemprichii* (Pearson product-moment correlation, *r* = 0.649, *p* = 0.001; figure 3*b*; electronic supplementary material, table S3) and *S. pistillata* (Pearson product-moment correlation, *r* = 0.446, *p* = 0.011; figure 3*c*; electronic supplementary material, table S3).

No relationships were found between *nirS* to *nifH* gene abundance ratios and Symbiodiniaceae cell densities for both corals combined (linear regression, *F* = 2.61, *r* = 0.279, *p* = 0.116; figure 3*d*), or per single species; *A. hemprichii* (linear regression, *F* = 1.35, *r* = 0.264, *p* = 0.260; figure 3*e*) and *S. pistillata* (linear regression, *F* = 2.21, *r* = 0.409, *p* = 0.165; figure 3*f*).

## 4. Discussion

Coral-associated microbial N cycling still remains an understudied, but arguably very important aspect of coral holobiont functioning as it may be a source or sink of bioavailable N [9,40]. Here, we assessed the proportional dynamics of two antagonistic N cycling pathways, i.e. denitrification and diazotrophy, via means of the relative abundance of key genes, in two common central Red Sea coral species (figure 1*a*) in a seasonal resolution. To this end, proportional abundances of the functional marker gene *nirS*, as a proxy for denitrification [21], were calculated in relation to the functional marker gene *nifH*, as a proxy for diazotrophy [20] (figure 1*b*). Importantly, the *nirS* to *nifH* gene abundance ratios presented in this study are not based on absolute, but relative, abundances of each respective marker gene. Consequently, this approach does not allow for any conclusion regarding the absolute abundance of marker genes, or, ultimately, absolute abundances of either denitrifiers or diazotrophs. Rather, changes in the ratio may be interpreted as a proxy for a shift in the relative abundance of denitrifying in relation to N_2_-fixing prokaryotes and serve as a proxy for the relative prevalence of the associated processes/pathways [20,21]. In this light, increasing ratios may reflect an increase in denitrifying microbes and/or a decrease in N_2_-fixing microbes and vice versa. Using this approach, we were able to characterize the seasonal dynamics of microbial N cycling in central Red Sea corals.

### 4.1. Seasonal patterns and environmental drivers of denitrification and N_2_ fixation potential in corals

The *nirS* to *nifH* gene abundance ratios followed a very similar pattern in both coral species across seasons (figure 1*b*). Autumn was characterized by the highest and spring by the lowest *nirS* to *nifH* gene abundance ratios, regardless of species. While relative gene abundances of both marker genes (i.e. *nirS* and *nifH*) do not allow any direct conclusion regarding activities of associated biological processes, previous studies using corals from the same location showed that relative abundances of marker genes correlated with denitrification and N_2_ fixation rates under these environmental conditions [20,21]. Consequently, the observed patterns of *nirS* to *nifH* gene abundance ratios may translate into similar seasonal patterns for associated denitrification to N_2_ fixation activities. In this light, the similarity of the seasonal patterns found in both coral species suggests that the functional niche occupied by different N cycling microbes may be very similar and highly responsive to changing environmental conditions.

Among all investigated environmental parameters, nitrate (the most significant contributor to DIN throughout all four investigated seasons; electronic supplementary material, figure S1) and DIN concentrations showed a moderate correlation with relative *nirS* to *nifH* gene abundance ratios across coral species (electronic supplementary material, table S3). As the substrate for denitrifiers, nitrate may directly stimulate denitrification activity [22,25]. Likewise, increased nitrate and/or ammonium concentrations have been shown to depress diazotroph activity [26–29,41]. The observed patterns in relative *nirS* to *nifH* gene abundance ratios may, thus, be the direct consequence of increased environmental N availability in the coral holobiont.

The notion of seasonally changing N availability driving patterns in ratios of prokaryotic N cycling functional groups within coral holobionts is corroborated by the patterns of Symbiodiniaceae cell densities observed in both coral species. Similar to *nirS* to *nifH* gene abundance ratios, Symbiodiniaceae cell densities exhibited strong seasonal differences, comparable to Symbiodiniaceae cell densities of conspecifics originating from the Red Sea [21,40,42,43], that positively correlated with environmental nitrate and DIN concentrations (figure 3*b*,*c*; electronic supplementary material, table S3). As the coral holobionts’ internal N concentrations were not measured in the present study, it is unknown whether environmental N was reflected by bioavailable N availability within the holobionts. However, Symbiodiniaceae population densities are known to be governed by N availability in the stable coral–algae symbiosis [44–46], suggesting that environmental N availability was closely linked with N availability within the coral holobiont in the present study as previously observed in *ex situ* studies [47–49].

### 4.2. Dynamics of N cycling microbes as a buffer against seasonal changes in environmental N availability

Limited N availability is critical to coral holobiont functioning as it limits population growth of Symbiodiniaceae *in hospite* and maintains high rates of translocation of photosynthetic carbon (C) to the host [8–10,46]. Seasonal or anthropogenically driven increases in environmental N availability may consequently stimulate Symbiodiniaceae proliferation, thereby disrupting or reducing organic C translocation to the host, ultimately posing a threat to overall coral holobiont functioning [11,50]. Yet, coral holobionts manage to thrive in highly dynamic environments with considerable temporal and spatial variations in N availability [14,15,22,51]. The positive correlation of Symbiodiniaceae densities and environmental N availability in the present study suggests that the coral hosts may not have been able to fully maintain stable N availability within the holobiont. In this light, the shift in relative *nirS* to *nifH* gene abundance ratios in response to the availability of environmental N could prove beneficial to the coral holobiont by partly stabilizing N levels in the host relative to environmental fluctuations. As such, during periods of low N availability (e.g. spring), low relative *nirS* to *nifH* gene abundance ratios probably imply reduced denitrification and increased N_2_ fixation activity. Likewise, during periods of high N availability (e.g. autumn), high relative *nirS* to *nifH* gene abundance ratios probably reflect increased denitrification and reduced N_2_ fixation activity. If indeed translatable to corresponding prokaryotic activity, the observed dynamics in functional N cycling gene abundance ratios may, thus, directly support coral holobiont functioning [9]. Specifically, the interplay of denitrifiers and N_2_-fixers may support the removal of excess N, while providing access to new bioavailable N in times of low environmental N availability [9,18]. While these processes may evidently have been insufficient for the stabilization of N availability within the holobiont, as shown in the present study, they may be directly assisting the coral host in regulating Symbiodiniaceae populations.

### 4.3. Future research directions

The present study adds to a rapidly growing body of research, highlighting the functional importance of N cycling microbes in coral holobiont functioning. Deciphering the interactions between N cyclers, not just those capable of N_2_ fixation and denitrification but also potentially important N cyclers capable of e.g. nitrification and ANAMMOX, and other coral holobiont members promises to advance our understanding of coral holobiont functioning in light of environmental conditions and anthropogenically driven change. While combined molecular (sequencing, real-time PCR) and physiological approaches haven proven powerful tools to study N cycling properties of coral holobionts, future studies should aim to address the identification and localization of the main microbial players along with accurate quantification of metabolic interactions with other holobiont members. In this light, fluorescence *in situ* hybridization, as well as nanoscale secondary ion mass spectrometry (NanoSIMS) techniques, may allow for an integrated and functional understanding of metabolic interactions in light of their localization within the coral holobiont.

## Data accessibility

The supporting data are provided in the electronic supplementary material and in the Dryad Digital Repository: https://doi.org/10.5061/dryad.rjdfn2z8k [52].

## References

[1] Rohwer F, Seguritan V, Azam F, Knowlton N. 2002 Diversity and distribution of coral-associated bacteria. Mar. Ecol. Prog. Ser. **243**, 1-10. (10.3354/meps243001)

[2] Falkowski PG, Dubinsky Z, Muscatine L, Porter JW. 1984 Light and the bioenergetics of a symbiotic coral. Bioscience **34**, 705-709. (10.2307/1309663)

[3] Robbins SJ et al. 2019 A genomic view of the reef-building coral *Porites lutea* and its microbial symbionts. Nat. Microbiol. **4**, 2090-2100. (10.1038/s41564-019-0532-4) 31548681

[4] Falkowski PG. 1997 Evolution of the nitrogen cycle and its influence on the biological sequestration of CO_2_ in the ocean. Nature **387**, 272-275. (10.1038/387272a0)

[5] Wang JT, Douglas AE. 1999 Essential amino acid synthesis and nitrogen recycling in an alga-invertebrate symbiosis. Mar. Biol. **135**, 219-222. (10.1007/s002270050619)

[6] LaJeunesse TC, Parkinson JE, Gabrielson PW, Jeong HJ, Reimer JD, Voolstra CR, Santos SR. 2018 Systematic revision of Symbiodiniaceae highlights the antiquity and diversity of coral endosymbionts. Curr. Biol. **28**, 2570-2580.e6. (10.1016/j.cub.2018.07.008) 30100341

[7] Muscatine L. 1990 The role of symbiotic algae in carbon and energy flux in reef corals. In Coral reefs (ed. Z Dubinsky), pp. 75-87. Amsterdam, The Netherlands: Elsevier.

[8] Béraud E, Gevaert F, Rottier C, Ferrier-Pagès C. 2013 The response of the scleractinian coral *Turbinaria reniformis* to thermal stress depends on the nitrogen status of the coral holobiont. J. Exp. Biol. **216**, 2665-2674. (10.1242/jeb.085183) 23531826

[9] Rädecker N, Pogoreutz C, Voolstra CR, Wiedenmann J, Wild C. 2015 Nitrogen cycling in corals: the key to understanding holobiont functioning? Trends Microbiol. **23**, 490-497. (10.1016/j.tim.2015.03.008) 25868684

[10] Dubinsky Z, Stambler N. 1996 Marine pollution and coral reefs. Glob. Chang. Biol. **2**, 511-526. (10.1111/j.1365-2486.1996.tb00064.x)

[11] Pogoreutz C, Rädecker N, Cárdenas A, Gärdes A, Voolstra CR, Wild C. 2017 Sugar enrichment provides evidence for a role of nitrogen fixation in coral bleaching. Glob. Chang. Biol. **23**, 3838-3848. (10.1111/gcb.13695) 28429531

[12] Wiedenmann J, D'Angelo C, Smith EG, Hunt AN, Legiret F-E, Postle AD, Achterberg EP. 2013 Nutrient enrichment can increase the susceptibility of reef corals to bleaching. Nat. Clim. Chang. **3**, 160-164. (10.1038/nclimate1661)

[13] Tilstra A, Bednarz VN, Cardini U, van Hoytema N, Al-Rshaidat MMD, Wild C. 2017 Seasonality affects dinitrogen fixation associated with two common macroalgae from a coral reef in the northern Red Sea. Mar. Ecol. Prog. Ser. **575**, 69-80. (10.3354/meps12206)

[14] Roth F, Saalmann F, Thomson T, Coker DJ, Villalobos R, Jones BH, Wild C, Carvalho S. 2018 Coral reef degradation affects the potential for reef recovery after disturbance. Mar. Environ. Res. **142**, 48-58. (10.1016/j.marenvres.2018.09.022) 30274715

[15] D'Angelo C, Wiedenmann J. 2014 Impacts of nutrient enrichment on coral reefs: new perspectives and implications for coastal management and reef survival. Curr. Opin. Environ. Sustain. **7**, 82-93. (10.1016/j.cosust.2013.11.029)

[16] Cardini U, Bednarz V, van Hoytema N, Rovere A, Naumann M, Al Rshaidat M, Wild C. 2016 Budget of primary production and dinitrogen fixation in a highly seasonal Red Sea coral reef. Ecosystems **19**, 771-785. (10.1007/s10021-016-9966-1)

[17] Lapointe BE, Brewton RA, Herren LW, Porter JW, Hu C. 2019 Nitrogen enrichment, altered stoichiometry, and coral reef decline at Looe Key, Florida keys, USA: a 3-decade study. Mar. Biol. **166**, 108. (10.1007/s00227-019-3538-9)

[18] Cardini U, Bednarz VN, Naumann MS, van Hoytema N, Rix L, Foster RA, Al-Rshaidat MMD, Wild C. 2015 Functional significance of dinitrogen fixation in sustaining coral productivity under oligotrophic conditions. Proc. R. Soc. B **282**, 20152257. (10.1098/rspb.2015.2257) PMC4650168 26511052

[19] Lema KA, Willis BL, Bourne DG. 2012 Corals form characteristic associations with symbiotic nitrogen-fixing bacteria. Appl. Environ. Microbiol. **78**, 3136-3144. (10.1128/AEM.07800-11) 22344646 PMC3346485

[20] Pogoreutz C, Rädecker N, Cárdenas A, Gärdes A, Wild C, Voolstra CR. 2017 Nitrogen fixation aligns with *nifH* abundance and expression in two coral trophic functional groups. Front. Microbiol. **8**, 1187. (10.3389/fmicb.2017.01187) 28702013 PMC5487474

[21] Tilstra A, El-Khaled YC, Roth F, Rädecker N, Pogoreutz C, Voolstra CR, Wild C. 2019 Denitrification aligns with N_2_ fixation in Red Sea corals. Sci. Rep. **9**, 19460. (10.1038/s41598-019-55408-z) 31857601 PMC6923481

[22] El-Khaled Y, Roth F, Tilstra A, Rädecker N, Karcher D, Kürten B, Jones B, Voolstra C, Wild C. 2020 In situ eutrophication stimulates dinitrogen fixation, denitrification, and productivity in Red Sea coral reefs. Mar. Ecol. Prog. Ser. **645**, 55-66. (10.3354/meps13352)

[23] Siboni N, Ben-Dov E, Sivan A, Kushmaro A. 2008 Global distribution and diversity of coral-associated Archaea and their possible role in the coral holobiont nitrogen cycle. Environ. Microbiol. **10**, 2979-2990. (10.1111/j.1462-2920.2008.01718.x) 18707612

[24] Kimes NE, Van Nostrand JD, Weil E, Zhou J, Morris PJ. 2010 Microbial functional structure of Montastraea faveolata, an important Caribbean reef-building coral, differs between healthy and yellow-band diseased colonies. Environ. Microbiol. **12**, 541-556. (10.1111/j.1462-2920.2009.02113.x) 19958382

[25] Zumft WG. 1997 Cell biology and molecular basis of denitrification. Microbiol. Mol. Biol. Rev. **61**, 533-616. (10.1016/j.sbspro.2014.08.122) 9409151 PMC232623

[26] Koop K et al. 2001 ENCORE: the effect of nutrient enrichment on coral reefs. Synthesis of results and conclusions. Mar. Pollut. Bull. **42**, 91-120. (10.1016/S0025-326X(00)00181-8) 11381890

[27] Cardini U, Bednarz VN, Foster RA, Wild C. 2014 Benthic N_2_ fixation in coral reefs and the potential effects of human-induced environmental change. Ecol. Evol. **4**, 1706-1727. (10.1002/ece3.1050) 24967086 PMC4063469

[28] Fay P. 1992 Oxygen relations of nitrogen fixation in cyanobacteria. Microbiol. Rev. **56**, 340-373. 1620069 10.1128/mr.56.2.340-373.1992 PMC372871

[29] Knapp AN. 2012 The sensitivity of marine N_2_ fixation to dissolved inorganic nitrogen. Front. Microbiol. **3**, 374. (10.3389/fmicb.2012.00374) 23091472 PMC3476826

[30] Tilstra A, Pogoreutz C, Rädecker N, Ziegler M, Wild C, Voolstra CR. 2019 Relative diazotroph abundance in symbiotic Red Sea corals decreases with water depth. Front. Mar. Sci. **6**, 372. (10.3389/fmars.2019.00372)

[31] Michotey V, Méjean V, Bonin P. 2000 Comparison of methods for quantification of cytochrome *cd*_1_-denitrifying bacteria in environmental marine samples. Appl. Environ. Microbiol. **66**, 1564-1571. (10.1128/AEM.66.4.1564-1571.2000) 10742243 PMC92024

[32] Gaby JC, Buckley DH. 2012 A comprehensive evaluation of PCR primers to amplify the *nifH* gene of nitrogenase. PLoS ONE **7**, e42149. (10.1371/journal.pone.0042149) 22848735 PMC3405036

[33] Pfaffl MW. 2001 A new mathematical model for relative quantification in real-time RT-PCR. Nucleic Acids Res. **29**, e45. (10.1093/nar/29.9.e45) 11328886 PMC55695

[34] Lavy A, Eyal G, Neal B, Keren R, Loya Y, Ilan M. 2015 A quick, easy and non-intrusive method for underwater volume and surface area evaluation of benthic organisms by 3D computer modelling. Methods Ecol. Evol. **6**, 521-531. (10.1111/2041-210X.12331)

[35] Gutierrez-Heredia L, Benzoni F, Murphy E, Reynaud EG. 2016 End to end digitisation and analysis of three-dimensional coral models, from communities to corallites. PLoS ONE **11**, e0149641. (10.1371/journal.pone.0149641) 26901845 PMC4763093

[36] Roth F et al. 2021 High summer temperatures amplify functional differences between coral- and algae-dominated reef communities. Ecology **102**, 1-15. (10.1002/ecy.3226) PMC7900985 33067806

[37] Clarke KR, Gorley RN. 2006 PRIMER v6: users manual/tutorial, pp. 1–192.

[38] Anderson MJ. 2001 A new method for non-parametric multivariate analysis of variance. Austral. Ecol. **26**, 32-46. (10.1111/j.1442-9993.2001.01070.pp.x)

[39] Anderson MJ. 2017 Permutational multivariate analysis of variance (PERMANOVA). *Wiley StatsRef Stat. Ref. Online*, 1–15. (10.1002/9781118445112.stat07841)

[40] Rädecker N et al. 2021 Heat stress destabilizes symbiotic nutrient cycling in corals. Proc. Natl Acad. Sci. **118**, e2022653118. (10.1073/pnas.2022653118) 33500354 PMC7865147

[41] Roth F et al. 2020 High rates of carbon and dinitrogen fixation suggest a critical role of benthic pioneer communities in the energy and nutrient dynamics of coral reefs. Funct. Ecol. **34**, 1991-2004. (10.1111/1365-2435.13625)

[42] Evensen NR, Fine M, Perna G, Voolstra CR, Barshis DJ. 2021 Remarkably high and consistent tolerance of a Red Sea coral to acute and chronic thermal stress exposures. Limnol. Oceanogr. **66**, 1718-1729. (10.1002/lno.11715)

[43] Voolstra CR, Buitrago-López C, Perna G, Cárdenas A, Hume BCC, Rädecker N, Barshis DJ. 2020 Standardized short-term acute heat stress assays resolve historical differences in coral thermotolerance across microhabitat reef sites. Glob. Chang. Biol. **26**, 4328-4343. (10.1111/gcb.15148) 32567206

[44] Falkowski PG, Dubinsky Z, Muscatine L, McCloskey L. 1993 Population control in symbiotic corals: ammonium ions and organic materials maintain the density of zooxanthellae. Bioscience **43**, 606-611. (10.2307/1312147)

[45] Xiang T, Lehnert E, Jinkerson RE, Clowez S, Kim RG, Denofrio JC, Pringle JR, Grossman AR. 2020 Symbiont population control by host-symbiont metabolic interaction in Symbiodiniaceae-cnidarian associations. Nat. Commun. **11**, 108. (10.1038/s41467-019-13963-z) 31913264 PMC6949306

[46] Krueger T, Horwitz N, Bodin J, Giovani M-E, Escrig S, Fine M, Meibom A. 2020 Intracellular competition for nitrogen controls dinoflagellate population density in corals. Proc. R. Soc. B **287**, 20200049. (10.1098/rspb.2020.0049) PMC7126079 32126963

[47] Grover R, Maguer JF, Allemand D, Ferrier-Pages C. 2006 Urea uptake by the scleractinian coral *Stylophora pistillata*. J. Exp. Mar. Biol. Ecol. **332**, 216-225. (10.1016/j.jembe.2005.11.020)

[48] Grover R, Maguer J-F, Allemand D, Ferrier-Pagès C. 2003 Nitrate uptake in the scleractinian coral *Stylophora pistillata*. Limnol. Oceanogr. **48**, 2266-2274. (10.4319/lo.2003.48.6.2266)

[49] Grover R, Maguer J-F, Reynaud-Vaganay S, Ferrier-Pages C. 2002 Uptake of ammonium by the scleractinian coral *Stylophora pistillata*: effect of feeding, light, and ammonium concentrations. Limnol. Oceanogr. **47**, 782-790. (10.4319/lo.2002.47.3.0782)

[50] Vega Thurber RL, Burkepile DE, Fuchs C, Shantz AA, Mcminds R, Zaneveld JR, Thurber RLV, Burkepile DE, Fuchs C. 2014 Chronic nutrient enrichment increases prevalence and severity of coral disease and bleaching. Glob. Chang. Biol. **20**, 544-554. (10.1111/gcb.12450) 24277207

[51] Stuhldreier I, Sánchez-Noguera C, Roth F, Cortés J, Rixen T, Wild C. 2015 Upwelling increases net primary production of corals and reef-wide gross primary production along the pacific coast of Costa Rica. Front. Mar. Sci. **2**, 113. (10.3389/fmars.2015.00113)

[52] Tilstra A, Roth F, El-Khaled YC, Pogoreutz C, Rädecker N, Voolstra CR, Wild C. 2021 Data from: Relative abundance of nitrogen cycling microbes in coral holobionts reflects environmental nitrate availability. *Dryad Digital Repository*. (10.5061/dryad.rjdfn2z8k) PMC8170195 34109033

